# Oxalate of Lime, and Its Relations to Certain Forms of Neuralgia

**Published:** 1853-09

**Authors:** H. A. Johnson


*Oxalate of Lime, and its relations to certain forms of Neuralgia.* By H. A. Johnson, M. D.—We are indebted for what we know of this deposit mostly to Dr. Golding Bird. From his observations he was led to the conclusion that oxalate of lime occurs in more than one-third of the cases in connection with an excess of uric acid or urate of ammonia, that in all cases there is an excess of urea, and that it is frequently accompanied by an excess of the phosphates; he also thinks it probable, that the uric and oxalic diatheses are both produced by the same morbid influences.

Uric acid, as existing in normal urine, is, without doubt, derived from the nitrogenized tissues of the body, but when found in excess, it may usually be traced either to ingesta, which the juices of the stomach have not power to dissolve, or to a too rapid destruction of tissues under the influence of heat, &c., as in fevers and inflammations; can oxalic acid be traced to a similar source?

Dr. G. Bird has presented a very ingenious theory for the production of this acid from urea and uric acid, but there is generally an excess of one or both of these ingredients accompanying the oxalic deposits. Should we expect this to be the case, if the abnormal product was the result of a transformation of the urea and uric acid? It seems to me not. My own observations have been limited, but I have thought, from a careful study of quite a number of cases, that, while a temporary functional disease of the digestive organs or the introduction into the digestive tube of a large amount of food, difficult of perfect solution in the juices of the stomach, will generally give rise to uric acid deposits in the urine, chronic disease of the alimentary canal, whether functional or organic, will *more* generally be found to exist with the oxalic diathesis.

In quite a large number of instances in which I have observed the oxalate of lime in the urine, the patients have not only been affected with dyspepsia, but have also been subject to severe attacks of neuralgia. In the first few instances, the neuralgic pains were confined to the lower extremities, and I strongly suspected that they were produced by mechanical irritation of the vesical mucus membrane from the crystals of the oxalate with which the urine was loaded at the commencement of the attack, but which, towards the termination, were replaced by an excess of the phosphates.

I have since observed neuralgic pains in the face, superior extremities and in the chest, co-existing with oxaluria, and, after carefully studying a number of cases, it seems to me evident, that the neuralgia and the urinary deposits sustain to each other an intimate relation through a common cause, viz: the derangement of the digestive organs.

It is perhaps probable that oxalic acid, whether produced from malassimilated food, as I think, or, from a metamorphosis of urea and uric acid, may exist first in combination with ammonia, as Dr. G. Bird has suggested. If so, it is possible, that it may, instead of being selected by the kidneys and combining in those organs with lime, be precipitated in the tissues. The crystals of this new salt, many of them smaller than blood globules, and presenting sharp angles and edges, may thus, by mechanical irritation, act directly upon the suffering structures, producing that intense and indescribable pain, for controlling which, anodynes and narcotics have so little power.

Permit me to allude to my own personal experience. During the last few months I have been frequently annoyed by neuralgic pains, and always after eating freely of oranges. I had never been in the habit of using this fruit until during my recent visit south, but while in Natchez and New Orleans, it constituted almost my only diet. I also ate very freely of it during my return home.

It is to my mind an interesting fact, that the first neuralgic pain that

I ever experienced, so far as my memory serves me, was while in Natchez. Since my return I have frequently partaken of the fruit, and almost always with the same result, pains of a neuralgic character in my face, chest, knee, dorsum of the foot, &c. These facts induced me to institute the following experiment.

At 8 o'clock A. M., I breakfasted on beef steak, potatoes, corn bread and two eggs. After breakfast I walked two miles. At 11 A. M., the urine passed, was normal in color, specific gravity 1030. After standing, there was no deposit of any kind; on a careful microscopic examination, I was unable to detect a single crystal of the oxalate of lime. I then ate four large oranges; at 1 P. M., I dined on a small quantity of roast beef, and whortleberry and green currant pie. At 7 P. M., the urine passed was of straw color, specific gravity 1036. After standing 30 minutes, a sediment was thrown down, consisting mainly of oxalate of lime in very large beautiful crystals. I think I never saw a specimen of urine in which it existed in greater abundance, or in which the crystals were larger. It also contained urate of ammonia and an excess of urea. I placed some of it in a watch glass, and added strong nitric acid; in a few moments it was almost a solid mass from the crystals of nitrate of urea. The urine passed the next morning at 7 o'clock had a specific gravity of 1030, and contained an excess of urea and uric acid and epithelial scales. At 11 P. M., the urine was normal. I then ate four more large oranges, and went to bed. The urine passed at 7 o'clock the next morning was loaded with the oxalates. These two experiments, one upon the urine of food, and the other upon the urine of blood, seem to me to indicate: 1st, that oxalic acid may be produced from the ingesta; 2d, that oranges, and probably all fruits containing citric acid, may give rise to the oxalic diathesis.

The manner in which the transformation is effected is uncertain, nor is it a matter of much consequence. I venture, however, to present as a possible explanation the following formula:

| | C. | H. | O. | | C. | H. | O. | |
|---|---|---|---|---|---|---|---|---|
| 6 at. of cit. acid = | 24 | 18 | 30. | = | 12 | | 18 | = 6 at. oxalic acid. |
| | | | | | 12 | 10 | 10 | = 2 at. lactic acid. |
| | | | | | | 2 | 2 | = 2 at. of water. |
| | | | | | | 6 | | |

We have remaining six atoms of hydrogen, which, united with 2 at. of nitrogen, would produce 2 at. of ammonia.

What influence have the organic acids in causing neuralgia through the derangement which they produce in the performance of the digestive functions? Do the neuralgic pains depend upon mechanical irritation, as I think may be possible, or do they depend upon perverted nutrition of the nervous structures, as I think more probable? These are interesting subjects of inquiry, but I have not time to pursue them further. —*North Western Med. and Surgical Journal.*

